# Association between vascular endothelial growth factor receptor 2 rs11941492 C/T polymorphism and Chinese Han patients in rheumatoid arthritis

**DOI:** 10.1097/MD.0000000000018606

**Published:** 2019-12-27

**Authors:** Zhicheng Yang, Mingjie Wang, Ting Yan, Zhiyong Hu, Hui Zhang, Ruiping Liu

**Affiliations:** Department of Orthopedics, The Affiliated Changzhou No.2 People's Hospital of Nanjing Medical University, Changzhou, China.

**Keywords:** molecular epidemiology, polymorphism, rheumatoid arthritis, VEGFR2

## Abstract

The aim of the present study was to examine the association between vascular endothelial growth factor receptor 2 (*VEGFR2*) rs11941492 C/T polymorphism and rheumatoid arthritis (RA) risk in an eastern Chinese Han population. We examined *VEGFR2* rs11941492 C/T polymorphism in 615 RA patients and 839 controls in an East Chinese Han population. The power analysis was used for evaluating the reliability of the results. Genotyping was performed using a custom-by-design 48-Plex single nucleotide polymorphism scan Kit. Pooled odds ratios (ORs) and 95% confidence intervals (CIs) were calculated using logistic regression.

Our results indicated that *VEGFR2* rs11941492 C/T polymorphism (TT vs CC, *P* = .012, OR = 0.61, 95% CI = 0.41–0.89; TT vs CT + CC, *P* = .017, OR = 0.63, 95% CI = 0.43–0.92) was associated with a significantly decreased risk of RA. The power analysis showed that this study had a power of 98.5% to detect the effect of rs11941492 C/T polymorphism on RA susceptibility, assuming an OR of 0.61. After stratification analysis, a decreased risk of RA was associated with *VEGFR2* rs11941492 TT genotype (TT vs CC) among female patients (TT vs CC, *P* = .007, OR = 0.53, 95% CI = 0.33–0.84), older patients (Yr ≥55) (TT vs CC, *P* = .039, OR = 0.58, 95% CI = 0.35–0.97), C-reactive protein-positive patients, anti-cyclic citrullinated peptide antibody-negative patients, rheumatoid factor-positive patients (TT vs CT + CC, *P* = .015, OR = 0.60, 95% CI = 0.39–0.90), functional class III + IV patients, patients with a DAS28 of ≥3.20, and those with an erythrocyte sedimentation rate of <25. However, our results were obtained from only a moderate-sized sample. Studies with larger sample sizes in other ethnic populations are needed to confirm these results. The *VEGFR2* rs11941492 genotype is associated with decreased susceptibility to RA.

## 1 Introduction

Rheumatoid arthritis (RA) is a chronic destructive joint disease, and synovial angiogenesis is considered to be important in its pathogenesis.^[1]^ Angiogenesis, which occurs in the early stage of RA, is an important process in proliferative synovitis and is essential for the progression of the arthritic lesion.[^2^,^3^,^4^] However, the mechanisms that promote angiogenesis in RA are not clearly understood.^[5]^ Some studies have supported the important role of vascular endothelial growth factor (VEGF) in RA angiogenic processes.^[6]^ Once the autoimmune process of RA has been initiated, the joint inflammation may lead to relative hypoxia of synovial tissue because VEGF expression is potentiated in response to the hypoxic state in joints with RA.^[7]^

Two signal transduction pathways of the VEGF receptor have been identified: the tyrosine kinase receptor and non-receptor tyrosine kinases. The tyrosine kinase receptor contains vascular endothelial growth factor receptor 1(VEGFR1), VEGFR2, and VEGFR3.[^8^,^9^,^10^] VEGFR2 is the main VEGF functional receptor, of which 2 types have been studied: human VEGFR2, termed kinase inserted domain-containing receptor, and mouse VEGFR2, termed fetal liver kinase-1. VEGFR2 is an important mediator of endothelial cell proliferation in angiogenesis.[^11^,^12^] VEGFR2 is also involved in the changes in vascular permeability mediated by VEGF.[^13^,^14^] The combination of VEGFR2 and VEGF induces the formation of dimers and tyrosine phosphorylation, which leads to a series of changes of endothelial cells. In summary, modulation of VEGF/VEGFR signaling activity offers an attractive target for inhibition of aberrant angiogenesis.^[11]^ Based on these results, we hypothesized that VEGFR2 may be involved in the angiogenesis of RA.

Despite the possible importance of angiogenesis in RA, few studies investigated the association between *VEGFR2* gene polymorphism and RA risk. Only 1 study by Paradowska-Gorycka et al explored *VEGFR2* gene polymorphisms and protein levels in relation to susceptibility and severity of RA. They found that the *VEGFR2* rs1870377 A/T, and rs2305948 G/A polymorphisms were associated with an increased risk of RA, while *VEGFR2* rs2071559 T/C polymorphism was related to a decreased risk of RA.^[15]^ In addition, they observed that rs2305948 G/A polymorphism showed a significant positive association with the severity of RA.^[15]^ However, they did not explore the relationship between *VEGFR2* rs11941492 C/T polymorphism and RA risk. Thus, we designed this case-control study to assess whether *VEGFR2* rs11941492 C/T polymorphism was associated with RA susceptibility in an eastern Chinese Han population.

## 2 Patients and methods

### 2.1 Study subjects

We obtained approval for the study protocol from the Ethics Committee of Nanjing Medical University (Nanjing, China). All patients provided written informed consent before their participation.

Six hundred fifteen RA patients who fulfilled the criteria for RA set by the American College of Rheumatology (1987)^[16]^ were consecutively recruited from the Changzhou Second Hospital-Affiliated Hospital of Nanjing Medical University, the Changzhou First Hospital, and the Changzhou Traditional Chinese Medical Hospital, between September 2010 and October 2013. Individuals who were RA patients at least had 4 symptoms:

(1) Morning stiffness in and around joints at least 1 hour
(2) Soft tissue joint swelling observed by physician at least 3/14 joint groups
(3) Soft tissue joint swelling in a hand joint
(4) Symmetrical swelling of 1 joint area
(5) Rheumatoid nodule
(6) Rheumatoid factor (RF) by method positive in <5% normal population
(7) Radiograph changes on wrist/hands: erosions or juxta-articular osteoporosis.

The exclusion criteria were as following:

(1) Patients with other autoimmunological disease and were excluded from the study.
(2) Patients with neoplasmatic diseases or metabolic diseases were also not included in this study.

The controls were patients without RA, matched for age (±5 years) and sex, and recruited from the same institutions during the same time period; the majority of the controls were admitted to the hospitals for the treatment of trauma.

To obtain demographic and RA risk factor data, each patient was interviewed by 2 trained reviewers (Hui Zhang and Zhicheng Yang) using a pre-validated questionnaire. Following the interview, 2 mL of peripheral blood was collected from each patient.

Blood samples were collected using vacutainers, and transferred to test tubes containing ethylenediaminetetraacetic acid, using the QIAamp DNA Blood Mini Kit (Qiagen, Hilden, Germany). The concentration of the DNA obtained was about 30 ng/uL. Single nucleotide polymorphism (SNP) genotyping was performed using a custom-by-design 48-Plex SNP scan^TM^ Kit (Genesky Biotechnologies Inc, Shanghai, China) as previously described.^[17]^

### 2.2 Power analysis

To estimate the statistical power of our study design, we implemented a comprehensive power analysis using Genetic Power Calculator 33. The power of this study was calculated with a significant value of 0.05.^[18]^

### 2.3 Statistical analyses

Differences in the demographic characteristics, variables, and the genotypes of the *VEGFR2* rs11941492 C/T polymorphism variants were evaluated using a Chi-squared test or Student *t* test. Associations between *VEGFR2* rs11941492 C/T genotypes and risk of RA were estimated by computing odds ratios (ORs) and 95% confidence intervals (CIs) using logistic regression analyses. Pooled ORs and 95% CIs were calculated in allele model, dominant model, recessive model, homozygous model, and heterozygous models. The Hardy–Weinberg equilibrium (HWE) principle was tested by a goodness-of-fit Chi-squared test, to compare the observed and expected genotype frequencies among controls. Subgroup analyses were conducted by age, sex, functional class, DAS28, erythrocyte sedimentation rate (ESR), C-reactive protein (CRP) status, anti-cyclic citrullinated peptide antibody (ACPA) status, and RF status. All statistical analyses were performed using the SAS software package (ver. 9.1.3; SAS Institute, Cary, NC).

## 3 Results

### 3.1 Characteristics of the study population

The demographic and clinical characteristics of all subjects are summarized in Table 1. Subjects were adequately matched for age and sex. There were no significant differences in the distributions of age and gender status (*P* = .170 and *P* = .566, respectively). The mean age of RA patients was 54.51 ± 15.19 years and the mean age of healthy controls was 55.44 ± 10.80 years. Furthermore, some clinical parameters, including RF, ACPA, CRP, ESR, DAS28, functional class were also listed in the column of cases. The genotype distributions, of *VEGFR2* rs11941492 C/T in all subjects, are delineated in Table 2. The observed genotype frequencies, for polymorphisms in controls, were in HWE for *VEGFR2* rs11941492 C/T (*P* = .890).

**Table 1:**
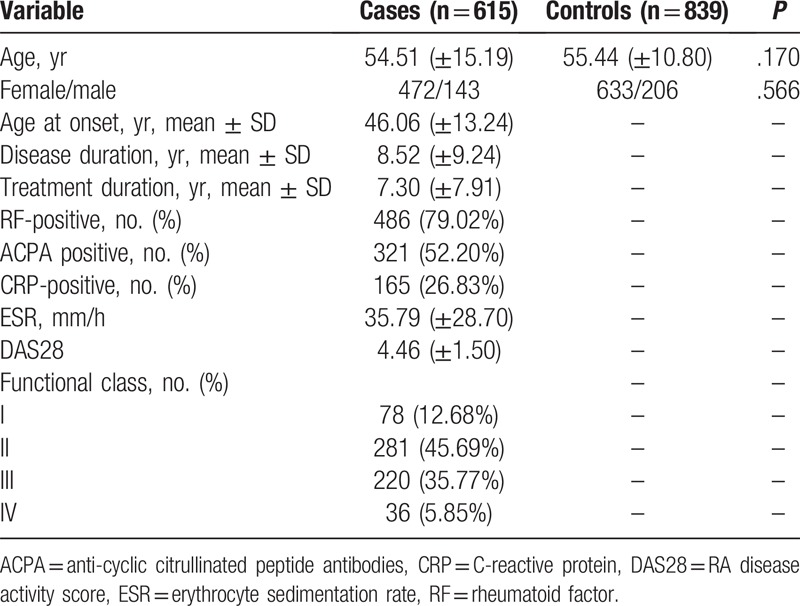
Patient demographics and risk factors in rheumatoid arthritis, all subjects.

**Table 2:**
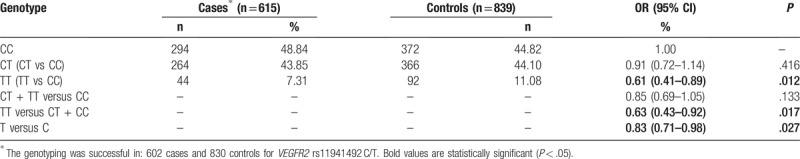
Logistic regression analysis of associations between *VEGFR2* rs11941492 C/T Polymorphisms and risk of rheumatoid arthritis.

### 3.2 Associations between VEGFR2 rs11941492 C/T polymorphisms and the risk of RA

Logistic regression analyses revealed that, when the *VEGFR2* rs11941492 CC homozygote genotype was used as the reference group, the TT genotype was associated with significantly decreased risk of RA (TT vs CC: OR = 0.61, 95% CI = 0.41–0.89, *P* = .012). The *VEGFR2* rs11941492 TT genotype is also associated with a decreased risk for RA compared TT genotype with CC + CT genotypes. (TT vs CC + TT: OR = 0.63, 95% CI = 0.43–0.92, *P* = .017) (Table 2). Our analysis also revealed that *VEGFR2* rs11941492 T allele was associated with significantly decreased risk of RA (T vs C: OR = 0.83, 95% CI = 0.71–0.98, *P* = .027) than the rs11941492 C allele in the Chinese Han population.

### 3.3 Stratification analyses of VEGFR2 rs11941492 C/T polymorphisms and the risk of RA

Stratification analyses were performed according to age, sex, RF status, DAS28 status, CRP status, ESR status, functional status, and ACPA status (Table 3). Following stratified analysis, a decreased risk of RA was associated with the *VEGFR2* rs11941492 TT genotype (TT vs CC) among female patients (OR = 0.53, 95% CI = 0.33–0.84, *P* = .007), older patients (Yr ≥55) (OR = 0.58, 95% CI = 0.35–0.97, *P* = .039), CRP-positive patients (OR = 0.51, 95% CI = 0.31–0.83, *P* = .007), ACPA-negative patients (OR = 0.59, 95% CI = 0.35–1.00, *P* = .048), RF-positive patients (OR = 0.56, 95% CI = 0.37–0.87, *P* = .009), functional class III + IV patients (OR = 0.55, 95% CI = 0.32–0.96, *P* = .035), patients with a DAS28 of ≥3.20 (OR = 0.61, 95% CI = 0.40–0.93, *P* = .022), and those with an ESR of <25 (OR = 0.54, 95% CI = 0.31–0.94, *P* = .030) (Table 3). The *VEGFR2* rs11941492 TT genotype (TT vs CT + CC) may decrease the risk of RA among female patients (OR = 0.54, 95% CI = 0.34–0.85, *P* = .008), CRP-positive patients (OR = 0.55, 95% CI = 0.34–0.89, *P* = .014), ACPA-negative patients (OR = 0.60, 95% CI = 0.36–0.99, *P* = .047), RF-positive patients (OR = 0.60, 95% CI = 0.39–0.90, *P* = .015), patients with a DAS28 of ≥3.20 (OR = 0.66, 95% CI = 0.44–0.99, *P* = .046), and those with an ESR of <25 (OR = 0.54, 95% CI = 0.31–0.92, *P* = .022) (Table 3).

**Table 3:**
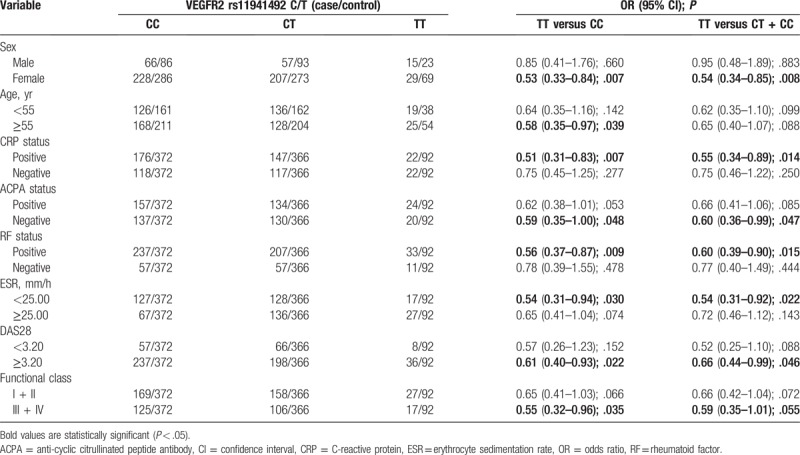
Stratified analyses between VEGFR2 rs11941492 C/T polymorphisms and the risk of rheumatoid arthritis.

## 4 Discussion

In the current case-control association study, we attempted to demonstrate an association between *VEGFR2* rs11941492 C/T gene polymorphism and susceptibility to RA in a Chinese Han population. Our study is the first time to investigate the association between *VEGFR2* rs11941492 C/T gene polymorphism and susceptibility to RA patients. Our present data suggest that the *VEGFR2* rs11941492 C/T genotype is associated with decreased susceptibility to RA. In stratification analysis, we also found a decreased risk of RA was associated with the *VEGFR2* rs11941492 TT genotype (TT vs CC) among female patients, older patients (Yr ≥55), CRP-positive patients, ACPA-negative patients, RF-positive patients, functional class III + IV patients, patients with a DAS28 of ≥3.20, and those with an ESR of <25. The *VEGFR2* rs11941492 TT genotype (TT vs CT + CC) may decrease the risk of RA among female patients, CRP-positive patients, ACPA-negative patients, RF-positive patients, patients with a DAS28 of ≥3.20, and those with an ESR of <25, which indicated *VEGFR2* rs11941492 C/T gene polymorphism was not associated with the severity and progression of RA. The *VEGFR2* gene, a member of the receptor-tyrosine kinases superfamily, is located at the 4q11–13 chromosomal region,^[19]^ whose encoded protein is considered to be the main receptor mediating VEGF signals in endothelial cells. One study revealed that the VEGFR signal-transduction process regulates the biological responses to VEGF.^[20]^ Therefore, the correlation between VEGF with VEGFR2 is important in angiogenesis.^[21]^ Furthermore, VEGFR2 accounts for the majority of the angiogenic and permeability-enhancing effects of VEGF.^[22]^ Angiogenesis plays an important role in the pathogenesis of various diseases, such as cancer, arthritis, and autoimmune conditions.[^23^,^24^,^25^,^26^]

Recently, several studies have reported expression of VEGFR2 on tumor cells.[^27^,^28^] VEGFR2 is one of the antigens that is selectively upregulated on activated tumor-associated endothelial cells.^[29]^ Tumor cells express VEGF, which binds to VEGFR2 on endothelial cells^[30]^; therefore, promoting tumor angiogenesis. Furthermore, the differential expression of VEGFR2 on tumor-associated endothelial cells is a potential target for antiangiogenic treatments in malignant tumors.^[31]^

Some studies have investigated the relationship between VEGFR2 and autoimmune diseases.[^25^,^26^] Vazgiourakis et al revealed that *VEGFR2* SNPs may contribute to systemic lupus erythematosus (SLE) pathogenesis by impairing VEGF signaling, and none of the SNPs analyzed were associated with increased susceptibility to SLE. Seabrook et al reported increased *VEGFR2* expression by both glial cells in the rim of the lesion and blood vessels in human multiple sclerosis (MS) lesions. As is the case in the above studies, the role of VEGFR2 in the angiogenesis of RA might be similar to that in SLE and MS.[^25^,^26^] RA is a chronic inflammatory and destructive autoimmune disease, the progression of which appears to rely on synovial angiogenesis.^[1]^ Angiogenesis was also recognized as an important event in the formation and progress of the synovial pannus in RA.^[32]^ VEGF, an angiogenic factor, was abundant in the synovium of RA patients.^[33]^ VEGF binds to VEGFR2, a tyrosine kinase receptor present on endothelial cell membranes, which may be related to angiogenesis in some diseases.^[34]^

Therefore, we hypothesized that variations in the *VEGFR2* gene may change the biological function of the corresponding protein and thus affect endothelial function in patients with RA. Paradowska-Gorycka et al first found that the *VEGFR2* gene polymorphisms showed significant association with the risk of RA, and *VEGFR2* rs2305948 SNP was associated with DAS-28 score, VAS score, number of swollen joints, mean value of CRP, suggesting an important role of *VEGFR2* gene polymorphisms in the severity of RA.^[15]^ In this study, we observed that TT genotype of rs11941492 C/T polymorphism was associated with a decreased risk of RA. In addition, TT genotype carriers showed a percentage of functional class III + IV and a DAS28 of ≥3.20, which was in line with the findings by Paradowska-Gorycka et al. However, we did not examine VEGFR2 serum levels in patients with RA and analyzed the association between *VEGFR2* gene polymorphisms and its concentration. Further studies in other ethnic populations are urgently needed to verify these results.

Several limitations of the present study should be addressed. First, due to our use of a hospital-based case-control design, selection bias was unavoidable. Second, the sample size of this study was not sufficiently large to evaluate low-penetrance effects of the SNPs. Third, our results should be interpreted with caution due to the potential influence of confounding factors for RA, such as environmental and inflammatory factors. Fourth, we could not collect the treatment information for further analysis.

In conclusion, our present data suggest that the *VEGFR2* rs11941492 genotype is associated with decreased susceptibility to RA. The *VEGFR2* rs11941492 genetic variant may play a protective role in the development of RA in our population, which is not associated with the severity and progression of RA. In stratification analysis, we also identified a decreased risk of RA associated with the *VEGFR2* rs11941492 TT genotype (TT vs CC, and TT vs CT + CC) among female, CRP-positive, ACPA-negative, and RF-positive patients, and in patients with a DAS28 of ≥3.20 or an ESR of <25. However, as the results were obtained from 1 moderate-sized sample of Chinese Han patients, larger sample sizes and well-designed studies with ethnically diverse populations are warranted to further evaluate the association between *VEGFR2* SNPs and RA susceptibility. These findings can provide the basis for future individualized diagnosis and prevention.

## Author contributions

**Conceptualization:** Zhiyong Hu.

**Data curation:** Ting Yan.

**Formal analysis:** Mingjie Wang.

**Investigation:** Hui Zhang.

**Methodology:** Zhicheng Yang.

**Supervision:** Ruiping Liu.

**Writing – original draft:** Zhicheng Yang.

**Writing – review and editing:** Ruiping Liu.
